# Surgical Hints

**Published:** 1902-02

**Authors:** 


### Surgical Hints.

*Surgical Needles.*—It is a good thing to remember that surgical needles require sharpening about as often as scalpels, and that the use of a hone and a little emery powder will restore to usefulness many needles in an apparently hopeless condition.

*Draining Tube.*—It is well to remember that a drainage tube is a foreign body, and hence an evil. Clean surgery and proper attention to hæmastosis reduce considerably the number of cases in which drainage is indicated, and it seems to be the tendency of the best surgeons to do the least draining.

*A poultice* is an excellent thing in its place, but there is no earthly reason for ever making it out of dirty vegetable matter. Gauze and absorbent cotton soaked in weak, warm antiseptic solutions, and covered with protective, are the only poultices allowable in modern clean surgery.

*In cases of severe hemorrhage* from the lungs, due to external wounds, the chest should be immediately opened, and a good-sized drainage tube inserted. This causes pneumo-thorax, and the pressure of air upon the lungs will greatly help in controlling the bleeding. The intro-venous injection of saline solution must also be immediately employed.

*In crushing accidents* in which the limbs have been caught in machinery it is very difficult to cleanse the wound properly, owing to the fact that the parts are much covered with grease, due to lubricating substances. Ordinary gasoline is an excellent thing wherewith to remove this grease; it causes no pain, dissolves away the grease, and leaves a clean surface upon which watery solutions of antiseptics can exert their full power.

*Children* who are prepared for operation must not be kept as long without food, prior to anesthesia, as is proper in adults. Children weaken rapidly from hunger, and it is best to give them easily digested food up to three or four hours before the operation. As in the majority of instances, they need not know that an operation is contemplated; there is none of the inhibitory effect upon digestion caused by fear that is often observed in adults.

*It is important* to remember that children, especially in crowded, poor districts, sometimes have empyema without even complaining of chills or showing a rise of temperature, and that the disease is often so insidious as to lead simply to general ill-health long before the parents became alarmed at the child's condition. Any child that has become gradually run down in health should be stripped and carefully examined for empyema when no other cause is evident.—*International Journal of Surgery,* December, 1900.

